# Diagnostic and Therapeutic Updates in Leptomeningeal Disease

**DOI:** 10.1007/s11912-023-01432-2

**Published:** 2023-05-31

**Authors:** Meaghan A. Roy-O’Reilly, Tyler Lanman, Amber Ruiz, David Rogawski, Brian Stocksdale, Seema Nagpal

**Affiliations:** Department of Neurology, Stanford Medicine, Palo Alto, CA 94305 USA

**Keywords:** Neuro-oncology, Leptomeningeal disease

## Abstract

**Purpose of Review:**

Leptomeningeal disease (LMD) is a devastating complication of advanced metastatic cancer associated with a poor prognosis and limited treatment options. This study reviews the current understanding of the clinical presentation, pathogenesis, diagnosis, and treatment of LMD. We highlight opportunities for advances in this disease.

**Recent Findings:**

In recent years, the use of soluble CSF biomarkers has expanded, suggesting improved sensitivity over traditional cytology, identification of targetable mutations, and potential utility for monitoring disease burden. Recent studies of targeted small molecules and intrathecal based therapies have demonstrated an increase in overall and progression-free survival. In addition, there are several ongoing trials evaluating immunotherapy in LMD.

**Summary:**

Though overall prognosis of LMD remains poor, studies suggest a potential role for soluble CSF biomarkers in diagnosis and management and demonstrate promising findings in patient outcomes with targeted therapies for specific solid tumors. Despite these advances, there continues to be a gap of knowledge in this disease, emphasizing the importance of inclusion of LMD patients in clinical trials.

## Introduction

Leptomeningeal disease (LMD) refers to the spread of malignant cells to the leptomeninges, subarachnoid space, and other cerebrospinal fluid (CSF) compartments. The majority of LMD arises from solid tumors originating outside the central nervous system (CNS), although primary CNS tumors can also infiltrate the leptomeningeal space. The rate of known LMD in cancer patients is approximately 5%, with case series suggesting an incidence of asymptomatic or undiagnosed LMD in up to 20% of solid-tumor patients at the time of autopsy [1].

The incidence of LMD varies by primary cancer type and is most commonly found in breast, lung, and melanoma patients [2–4]. The majority of patients with LMD have coexisting parenchymal brain metastases (BrM), but up to 42% of LMD is diagnosed in the absence of parenchymal involvement [5]. LMD has been associated with advanced malignancy and very poor prognosis, with median survival in historical cohorts ranging from 1 month without therapy to 3–6 months with radiation and chemotherapy [6, 7]. However, the era of targeted therapy has fundamentally changed the diagnostic and therapeutic landscape for BrM and LMD patients; some novel therapies have extended median survival in certain types of LMD beyond 1 year [8••].

In this review, we discuss the clinical presentation, pathogenesis, diagnosis, and management of LMD arising from systemic solid tumors, lymphoma/leukemias, and primary brain tumors. We highlight recent advances and discuss opportunities for progress in the coming years.

## Pathophysiology

LMD can be subdivided based on tumor of origin: solid tumors, hematological malignancies, or primary CNS tumors [9]. More recently, LMD has been further sub-divided into two radiographic categories: classical LMD (cLMD), consisting of diffuse enhancement of the leptomeninges, and nodular MD (nMD), defined as focal, extra-axial nodular enhancing lesions of the meninges or ependyma. Given this relatively recent sub-classification, the data discussed within this review may include patients with either cLMD or nMD [10].

Numerous mechanisms of metastatic spread have been proposed, including direct extension into the leptomeningeal or CSF space [11], along perineural or endoneurial tracks [12], or via hematogenous or lymphatic spread [13, 14]. The CSF represents a protected space, shielded from the systemic immune system and systemically administered treatment by the BBB and blood-CSF-barrier (B-CSF-B) [15, 16]. Once established in the leptomeningeal space, metastatic cells can float freely in the CSF or adhere to the leptomeninges [17].

Next-generation sequencing demonstrates significant genomic heterogeneity between primary tumor cells and LMD [18]. Matrix metalloproteinases, which degrade extracellular matrix and promote cell adhesion, have been shown to be elevated in the CSF of patients with LMD [19]. Vascular endothelial growth factor and stromal derived factor-1 are also elevated, where they are hypothesized to contribute to the hypoxia-dependent angiogenesis required for metastatic survival [20, 21]. LMD interacts with the immune system via complement component 3, causing BBB breakdown and facilitating the entry of growth-promoting factors into the CSF [22]. LMD cells secrete lipocalin-2, promoting neoplastic cell growth and immune evasion via iron sequestration [23••]. However, the molecular mechanisms of migration, survival, and proliferation of tumor cells in LMD remains largely unknown, partly due to the difficulty in creating disease models that accurately simulate the leptomeningeal microenvironment [17, 24]. Early rodent LMD models involved direct injection of cancer cells into the cisterna magna or subarachnoid space; it was later found that injection of cancer cells into the internal carotid artery was more emulative [25]. Novel approaches are being developed, including use of patient-derived CSF circulating tumor cell (CTC) lines and genetically engineered murine LMD tumor models [26–28].

## Clinical Symptomatology and Complications

The diffuse nature of LMD often leads to multiple noncontiguous lesions, rendering it challenging to diagnose [15]. Increased intracranial pressure (ICP) is a common manifestation, thought to be the result of the accumulation of malignant cells and protein in the CSF, which can result in communicating or non-communicating hydrocephalus [1, 29]. Symptoms of increased ICP include headache (often positional) with associated nausea and dizziness, gait impairment, bilateral or unilateral cranial nerve 6 palsy, cognitive changes, and papilledema [30]. Among patients with elevated ICP, some will experience plateau waves, marked by sudden paroxysmal (often positionally provoked) ICP elevations with consequent dramatic neurologic symptoms with quick onset/offset [31]. In select patients, treating hydrocephalus via CSF diversion or radiotherapy can lead to symptomatic improvement [30]. Of note, given the theoretical risk of diverting cancer cells into the peritoneal space, CSF diversion is typically reserved for patients who have favorable options for CNS-directed therapy.

Independent of ICP, LMD can cause an inflammatory response resulting in headache and nuchal rigidity via meningeal disruption [32]. LMD can also infiltrate the underlying cortex via Virchow-Robin spaces [33], leading to seizures (~ 22% of LMD patients) [34] and cognitive impairment (~ 93% of patients) [35]. Given that LMD has a predilection for the posterior fossa [36], cerebellar involvement leading to ataxia is common [37]. Malignant cells from LMD can also invade the cerebral arteries, leading to strokes [38].

LMD can affect any cranial nerve (CN), resulting in isolated or multiple cranial neuropathies. LMD most commonly affects CN II, III, VI, and VII [39], leading to visual impairment, diplopia, and/or facial weakness. Among LMD patients with CN involvement, 50% had multiple cranial neuropathies; this often serves as a diagnostic red flag in cancer patients who do not yet have an established LMD diagnosis [40]. In addition to cranial nerves, LMD commonly involves spinal nerves, with symptoms of weakness, numbness, or radicular pain in the affected distribution [41].

## Prognostication

Despite advances in the treatment of systemic cancer, the prognosis of LMD remains poor, with only 15% of patients surviving 1 year following diagnosis. Similar to parenchymal BrM, the primary cancer type is a strong prognostic factor in patients with LMD. LMD from hematologic malignancies tend to have improved survival compared solid tumor LMD [42]. Among solid tumor LMD patients, data suggests that targeted therapy for LMD patients with actionable mutations may translate to improved survival [8••]. LMD caused by primary CNS disease has worse prognosis compared to other solid malignancies, likely reflecting that LMD typically occurs late in the disease course after therapeutic options have been exhausted [43]. Good functional status (as measured by Karnofsky performance status, or KPS) at the time of diagnosis and administration of systemic or intrathecal (IT) chemotherapy are the most robust positive prognostic factors [44, 45], although selection bias may confound these results as those receiving therapy tend to have better functional status/prognosis than those who are not offered or decline treatment. Reported negative prognostic factors include age > 60, increased CSF albumin, increased CSF lactate [43] and increased serum LDH [45]. Recent data suggests that prognosis may differ between nMD and cLMD, with shorter overall survival seen in patients with cLMD. [46]

## Diagnostics

Due to its variable and often nonspecific presentation, LMD is frequently overlooked until symptoms become severe. The core diagnostic workup for suspected LMD includes clinical exam, CNS imaging, and CSF sampling. Older case series reported that neurologic symptoms were clinically apparent in all patients at LMD diagnosis [47–49]. However, autopsy series have shown that the incidence of asymptomatic or undiagnosed LMD is ~ 20% [1]. On gadolinium-enhanced magnetic resonance imaging (MRI) of the brain and spine, findings indicative of LMD include focal or diffuse leptomeningeal enhancement, subarachnoid nodules, and cranial or spinal nerve enhancement [50]. Some studies suggested that delayed enhanced FLAIR sequences may provide better visualization of superficial meningeal enhancement compared to traditional post-contrast T1-weighted scans [51, 52]. However, CNS imaging for LMD has limited sensitivity; in a cohort of patients with cytology-proven LMD, 37% did not demonstrate LMD on MRI, with false negatives occurring more often with hematologic malignancies (52%) than solid tumors (28%) [5]. The specificity of CNS imaging of LMD is also limited, given that meningeal enhancement can result from a variety of non-neoplastic processes, including infectious or aseptic meningitis.

Given the limitations of clinical and radiologic techniques, CSF analysis is integral to LMD diagnosis. While the pattern of high cell count, low glucose, and high protein in CSF has been considered “suggestive” of LMD, these abnormalities in isolation are relatively nonspecific [53]. CSF cytology has high specificity for solid tumor LMD and is often considered the diagnostic gold standard; however, the sensitivity of cytology is limited, due to low volume of malignant cells within the CSF and technical variability. In a retrospective study of patients with paired CSF cytology who later underwent autopsy, the sensitivity of a single CSF cytology analysis was 59% [54]. Due to the volume-dependent sensitivity of cytology, it is recommended to send at least 10 ml of CSF, with repeat CSF sampling indicated if initial cytology is negative [55]. In hematologic malignancies, suspected LMD is evaluated with both cytology and flow cytometry; the latter has increased sensitivity for LMD secondary to leukemia or lymphoma and only requires ~ 2 ml CSF due to a lower limit of detection [56, 57]. Unlike cytology, flow cytometry is also a quantitative measure that can be serially monitored as a measure of disease burden and treatment response. [58]

Emerging technologies in the diagnosis of LMD include soluble CSF biomarkers, circulating tumor cells (CTCs), and cell-free tumor DNA (cfDNA). Several candidate soluble CSF biomarkers for LMD have been identified, but their clinical utility is limited by poor sensitivity or specificity [59–63]. Several assays for detecting CTCs in serum have been adapted for use with CSF, offering several advances over traditional cytology including improved sensitivity, quantitative monitoring of disease burden, and the ability to identify targetable mutations [58, 64, 65]. CellSearch is a commercially available assay that identifies serum CTCs based on the epithelial cell adhesion molecule (EpCAM) and has been studied prospectively with CSF for the diagnosis of LMD [58, 66]. This technique is limited to cancers that express EpCAM (i.e., carcinomas) and will not detect tumors that have undergone epithelial-mesenchymal transition with loss of EpCAM expression [67]. Another commercially available assay for use in CSF, CNSide (BioCept) utilizes a proprietary panel including EpCAM, cytokeratin, and nonepithelial markers of CTCs to obviate these limitations [67, 68]. The CNSide assay is also able to identify targetable mutations by fluorescent in situ hybridization (FISH) or immunohistochemistry (IHC) [67]. These analyses may have important therapeutic implications when the CSF CTC molecular profile is discordant with previously sampled sites of disease.

Due to normal cell turnover, short fragments (< 200 bp) of cfDNA from healthy cells are released into circulating serum and CSF [69]. In malignancy, a small portion of cfDNA comes from tumor cells themselves. Modern sequencing technologies allow for reliable detection of these rare malignant cfDNA in CSF with sensitivity for LMD that appears superior to either cytology or MRI alone [70, 71]. Similar to CTCs, cfDNA can also identify targetable genetic abnormalities that may not have been present in either the primary tumor or plasma cfDNA [64]. ARUP laboratories created a stand-alone CSF cfDNA-based test for the *EGFR* T970M mutation, which is seen in NSCLC and causes resistance to first- and second-generation *EGFR*-targeted therapies. In parallel to the CTC assay, the aforementioned CNSide product also allows for detection of cfDNA and actionable mutations in *EGFR, BRAF,* and *KRAS* [68].

While CTC and cfDNA testing for serum samples have been available for nearly a decade, the utility of these tests in clinical decision making and outcomes is still being explored. Recent results from the DYNAMIC trial demonstrated that randomization of colon cancer patients to further chemotherapy based upon the presence of serum cfDNA reduced the use of adjuvant chemotherapy without reducing recurrence-free survival [72]. The BFAST study, while not randomized, utilized serum cfDNA alone to identify NSCLC patients with actionable ALK-positive mutations for targeted therapy, an approach helpful in cases with inadequate availability of tumor tissue for comprehensive molecular analysis [73]. In LMD, cfDNA levels in the CSF have been shown to correlate with clinical response to treatment [74], but studies examining the utility and efficacy of CSF cfDNA in making treatment decisions and impact on survival outcomes are lacking. The FORSEE trial is an ongoing prospective study which aims to utilize CNSide technology for LMD diagnosis and clinical monitoring alongside standard of care treatment and diagnostic monitoring (NCT05414123). While both CTC and cfDNA testing is currently commercially available, more evidence-based data in CSF testing is required to guide the use of these CSF liquid biopsy diagnostics in clinical practice.

## Treatment of Leptomeningeal Disease

Given the challenges of LMD diagnosis, it is not surprising that there are few prospective, randomized, controlled trials evaluating the treatment of LMD. Therefore, clinicians and patients approach treatment decisions with limited data, balancing side effects with potential benefits to symptom burden and survival. Surgery and radiation have primarily palliative roles that can improve quality of life in select patients. The use of systemic therapy has historically been hampered by limited CNS penetration and activity. However, with the emergence of novel, molecular targeting agents and immunotherapy, there has been an expansion of pharmacologic options associated with improved survival outcomes. These agents can be administered as monotherapy, in combination with one another, or in combination with radiation therapy and/or surgical intervention. Treatment decisions should be based on a thoughtful balance of underlying tumor histology, presence of molecular targets, clinical performance status, and patient preference given that current treatments for solid tumor LMD are not curative.

## Radiotherapy

Radiotherapy (RT) is frequently utilized in LMD for symptomatic relief, debulking, and improvement in CSF flow [75]. Several RT modalities are utilized in LMD, the selection of which is dependent upon patient characteristics and LMD distribution. The 2022 National Comprehensive Cancer Network (NCCN) guidelines recommend stereotactic radio surgery (SRS), involved-field radiotherapy (IFRT), or whole-brain radiotherapy (WBRT) for LMD along with concomitant systemic therapy and/or IT chemotherapy in patients with favorable features (KPS ≥ 60, no major neurologic deficits, minimal systemic disease, and reasonable systemic treatment options) [53]. For patients with unfavorable features, including those with bulky CNS disease, palliative IFRT can be considered [53].

WBRT is generally preferred in cases with significant meningeal disease or in patients with simultaneous BrM, although data on efficacy are mixed [75]. WBRT alone is suggested as an effective palliative treatment for LMD patients with low KPS [76], and a recent systematic meta-analysis demonstrated that WBRT in combination with chemotherapy in patients with higher KPS is associated with prolonged survival in breast cancer and mixed-histology studies [44, 77, 78]. However, a retrospective study of LMD secondary to NSCLC showed no overall survival benefit with WBRT [79]. Therefore, when considering WBRT, it is critical to consider the individual patient’s symptoms and potential palliative benefit, as well as the overall predicted radiotherapy responsiveness of the primary cancer. For example, at our center, we offer WBRT to patients with NSCLC experiencing cranial nerve deficits.

Craniospinal irradiation (CSI) has been utilized for palliative treatment in LMD for relief of disabling neurologic symptoms [80–82]; but remains less utilized than WBRT or focal radiation in part due to concern for significant systemic side effects including bone marrow suppression and enteritis [75]. NCCN guidelines recommend the use of CSI be reserved for selected patient populations, primarily with LMD related to leukemia or lymphoma [53]. Proton CSI is a radiotherapy technique that limits the off-target delivery of RT beyond the neuro-axis, resulting in a reduction in systemic side effects. A recent phase Ib study by Yang et al. utilizing proton CSI in LMD secondary to solid tumors demonstrated durable disease control in select patients with a lower toxicity profile compared to standard photon CSI [83]. A recent interim analysis of an ongoing Phase II trial by this group demonstrated a significant increase in progression free survival (PFS) in patients treated with proton CSI compared to photon IFRT [NCT04343573]. Unfortunately, the utility of proton CSI for LMD treatment is limited by cost and the relative scarcity of proton-capable centers.

Focal radiation, either IFRT or SRS, can be used to treat bulky and symptomatic LMD, with the goal of reducing symptoms from LMD itself and/or by relieving CSF obstruction [75]. SRS allows for the administration of focused, high dose radiation to the target while the minimizing radiation exposure to nearby healthy tissue. While SRS is less commonly utilized in LMD, small studies suggest that it may represent an effective option to delay the need for WBRT in select patients, or for use in patients with bulky recurrence following prior WBRT [84–86].

## Surgical Interventions

While surgical interventions play a minor role in the treatment of LMD, several neurosurgical procedures are commonly utilized in LMD patients to facilitate chemotherapy delivery and aid in the symptomatic management of hydrocephalus.

### Ventricular Access Devices

Chemotherapy and some biologics can be delivered directly into the CSF (intrathecally, or IT) using either serial lumbar punctures (LP) or intraventricular (ICV) administration. LP is less invasive than ICV administration and generally has a lower risk of infectious complications. However, when repeat LPs are required, the risk for LP-related complications such as CSF leak, headache, and bleeding increases over time [87]. Local toxicity from accidental injection into the epidural space can also occur [88, 89]. While more invasive than LPs, ICV administration has several advantages including higher CSF drug levels, a more uniform distribution within the subarachnoid space, and faster, less painful procedures [88, 89]. In breast cancer, ICV delivery is associated with better treatment response and OS compared to lumbar injection [88]. An alternative to the ventricular access device is the chemoport, a bulkier device that is secured to the skull and can be used for chemotherapy delivery and, if connected to a CSF drainage system, utilized for extraventricular drainage [90].

### Ventriculoperitoneal Shunt and Ventriculostomy

As discussed previously, LMD can result in either communicating or obstructive hydrocephalus, leading to increased ICP and potentially devastating neurologic sequelae. Select patients may be considered for placement of a ventriculoperitoneal shunt (VPS) for the treatment of LMD-related hydrocephalus [91]. Lin et al. found that in this population, patients treated with VPS had significant overall clinical improvement (41% was sustained longer than 6 months) and did not experience shunt infections [92]. In some cases, a third ventriculostomy for LMD-associated hydrocephalus can be performed as an alternative to VPS [91]. In our clinical practice, VPS and ventriculostomy are typically reserved for patients with further options for systemic therapy.

## Systemic Treatment

### Traditional Chemotherapy

The role and selection of traditional systemic chemotherapy in LMD is dependent on the primary tumor histology and the drug’s CNS activity. Systemic therapy may be indicated if concurrent, active systemic disease is present [89]. There are several key benefits to systemic chemotherapy including ease of administration, impact on non-CNS disease and the ability of primary oncology teams to manage use [93]. Across all histological subtypes, no single systemic therapy has shown prolonged benefit with LMD. However, there are reports of survival benefit with systemic chemotherapy in select cancer types [43, 89, 94]. BBB-penetrating cytotoxic agents include high-dose methotrexate, capecitabine, temozolomide, lomustine, pemetrexed, and topotecan [89, 93].

### Targeted Therapy

In the past two decades, there has been an explosion of therapeutic agents for patients with cancers that harbor specific molecular targets. Both small molecule inhibitors and antibodies targeting cancer epitopes have activity in BrM and have made their way into the treatment paradigm for LMD. We will review several of these in detail, acknowledging that data supporting their use is often difficult to find, appearing only in supplemental materials or included as small exploratory cohorts. Furthermore, patients with LMD are often entirely excluded from clinical trials, so clinicians cautiously employ these targeted agents in LMD patients by virtue of extrapolation from data in parenchymal BM. Regardless, the agents reviewed below represent only a portion of those that are available and in the pipeline. Not covered here but emerging as important targeted therapies with CNS activity are small molecules inhibiting PARP, NTRK, RET, KRAS, and Met.

### Trastuzumab

Trastuzumab, a monoclonal antibody targeting HER2 receptors, has markedly changed clinical outcomes and survival in HER2 + breast cancer. There are several reports of significant delay of time to development of BrM in those who received systemic trastuzumab; however, a meta-analysis which included four randomized trials found that adjuvant systemic trastuzumab was associated with increased incidence of intracranial metastasis at time of first recurrence with a relative risk of 1.35 [95–97]. While the activity of single agent systemic trastuzumab in LMD is limited, there is emerging evidence that trastuzumab in combination therapy or as an antibody–drug conjugate may provide additional benefit in the prevention and management of LMD. Exploratory analysis of the phase III CLEOPATRA trial demonstrated that pertuzumab, in addition to trastuzumab and paclitaxel, was associated with delayed onset of CNS metastases in HER2 + breast cancer [98, 99]. In the Phase II PATRICIA study, HER2-positive metastatic breast cancer patients with CNS metastasis treated with pertuzumab plus high-dose trastuzumab were found to have a modest CNS objective response rate, with a majority of patients experiencing clinical benefit at 4 months [100].

Trastuzumab emtansine (T-DM1) is an anti-body drug conjugate linking trastuzumab to a cytotoxic anti-microtubule agent; it is currently utilized as second line therapy in HER2 + breast cancer. An exploratory analysis of 126 BrM patients on the KAMILLA study demonstrated a best ORR of 21.4%, clinical benefit of 42.9% and in the 67 patients with previously non-irradiated BrM, a response rate of 49.3% [101]. Two clinical trials evaluating trastuzumab deruxtecan, another antibody–drug conjugate, in HER2 + breast cancer with BM have shown hopeful results, with overall intracranial response rates ranging from 46.2 to 73.3% [102, 103]. These data refute the commonly held notion that antibody based drugs won’t have CNS activity due to an inability to penetrate the BBB. While these studies did not explicitly include LMD patients, a small case series of 6 patients with HER2 + LMD treated w T-Dx with a median overall survival (mOS) of 12.5 months suggests this is a viable treatment option for patients with HER2 + breast LMD [104]. The recent approval of TDx for HER2 + NSCLC makes this an option for non-breast CA patients, as well.

### Tyrosine Kinase Inhibitors

Small-molecule oral tyrosine kinase inhibitors (TKIs) have been increasingly utilized in the management of metastatic disease, particularly HER2-positive breast cancer and EGFR-mutant NSCLC. The CNS activity of these oral TKIs have been directly evaluated in several studies, both in the setting of untreated and progressive metastatic disease. Nearly a decade ago, the LANDSCAPE trial reported a high-level of CNS activity (CNS OR of 66% in untreated BrM) with lapatinib, a dual TKI with EGFR and HER2 directed activity [105]. Neratinib, an irreversible pan-HER TKI, has also demonstrated benefit in preventing CNS metastases in HER2 + breast cancer patients [105–107]. In 2020, the FDA-approved tucatinib, a highly selective HER2 inhibitor, with a label that included BrM. In a pre-planned exploratory analysis of 291 patients in the HER2CLIMB trial with BrM, tucatinib was associated with increased CNS progression-free survival (PFS, 9.9 months vs 4.2 months), and increased median time from BrM to secondary progression or death (7.6 months vs. 3.1 months) [108]. Patients with active BrM experienced a median OS of 20.7 months vs 11.6 months in the tucatinib vs control arms respectively [108]. While this study explicitly excluded patients with LMD, a small single center phase 2 that enrolled 17 HER2 + LMD patients demonstrated a CNS PFS of 6.9 months and mOS of 11.9 months [109]. The HER2CLIMB regimen offers another active, systemic therapy for patients with HER2 + LMD.

Like HER2 + breast CA, the high incidence of BrM in EGFRm NSCLC has led to a special emphasis on evaluating CNS activity earlier in drug development. This is especially true of osimertinib, a 3^rd^ generation, irreversible EGFR-TKI. In phase I and II studies, osimertinib demonstrated benefit in LMD secondary to EGFR-mutated NSCLC [8••, 110–113]. The phase I trial, BLOOM, evaluated osimertinib in patients with advanced EGFR-mutated NSCLC and LMD who progressed on prior TKI therapy; osimertinib showed both systemic and intracranial efficacy with a median PFS of 8.6 months and median OS of 11 months [113]. The efficacy of osimertinib in LMD was further supported by a phase II, multi-center study in which Park et al. reported a median PFS of 8 months and OS of 13.3 months [8••]. A retrospective review of the AURA studies, which evaluated the safety and efficacy of osimertinib in EGFR T790M-positive NSCLC, found a clinical benefit in those with LMD, noting a leptomeningeal ORR of 55%, median PFS of 11.1 months, and OS of 18.8 months [110]. A multi-institutional retrospective review assessed the efficacy of osimertinib dose escalation for CNS progression of EGFR-mutated NSCLC, reporting a median duration of CNS disease control of 3.8 months and median OS of 14.8 months in those with osimertinib alone [111]. Interestingly, patients with an LMD-only pattern of progression experienced a prolonged duration of CNS control compared to those with a parenchymal-only progression [111]. Though modest, there is a suggestion that dose escalation of osimertinib 80 mg to 160 mg daily may provide additional clinical benefit either alone or in combination therapy in the setting of progressive LMD due to EGFR-mutated NSCLC. Therefore, if a patient is osimertinib naïve at the time of LMD development, this is our treatment of choice. For patients who progress on osimertinib but retain the T790m mutation in the CSF, we consider dose escalation. If patents progress on osimertinib and the T790m mutation is no longer present in the CSF, but there is clear evidence of systemic disease control, we may add a systemic chemotherapy, such as pemetrexed.

### ALK Inhibitors

Anaplastic lymphoma kinase (ALK) inhibitors have proven beneficial in the treatment of ALK-rearranged NSCLC. Multiple iterations of the ASCEND trial have previously examined the ALK inhibitor ceretinib in NSCLC patients with and without BrM, demonstrating significant intracranial and extracranial responses in both heavily pre-treated and treatment-naïve patients [114–118]. Although prior ASCEND trials largely excluded patients with known LMD, the recent ASCEND-7 trial specifically examined the efficacy of ceretinb in patients with BrM, in addition to the inclusion of a dedicated study arm focused on patients with known LMD [119]. The results from this study demonstrated significant intracranial and extra-cranial responses patients with ALK-rearranged NSCLC with BrM, with more significant responses seen in ALK inhibitor naïve patients compared to those with prior ALK inhibitor treatment (47.5% vs. 33.3%). Within the LMD arm, the median PFS was 5.2 months with median OS of 7.2 months, which was felt to be increased compared to historical survival data [119]. Intracranial responses have also been reported in patients treated with the ALK inhibitors loratinib [120, 121], brigatinib [122, 123], and alectinib [124–126]. Unfortunately, symptomatic BrM and LMD patients remain excluded from most of these studies, so clinical use still requires extrapolation from these data. Taken together, these studies demonstrate the efficacy of that ALK inhibitors the treatment of intracranial ALK-rearranged NSCLC, and warrant consideration of their use as first and second line treatments in patients with the ALK driver mutation. These collective studies also once again highlight the critical gap in the treatment of LMD secondary to the widespread exclusion of these patients from clinical trials.

### CDK4/6 Inhibitors

Cyclin-dependent kinase 4 and 6 (CDK4/6) inhibitors (palbocliclib, ribocliclib, abemaciclib) have shown a clinical benefit in PFS and mOS in combination with hormone therapy for hormone receptor (HR) positive, HER2- breast cancer [127]. Abemaciclib has been shown to have good CNS penetration in clinical and preclinical studies [127–129]. In a non-randomized, phase II study evaluating the intracranial response in patients with metastatic HR + breast cancer with intracranial disease, a sub cohort of patients with LMD secondary to HR + , HER2- breast cancer receiving abemaciclib experienced median PFS of 5.9 months, mOS of 8.4 months and demonstrated an intracranial clinical benefit rate of 24% [130]. This modest benefit may be a useful option for patients who want to avoid the use of radiation or intrathecal therapies.

### BRAF Inhibitors

In addition to breast and lung cancer, melanoma also has a predilection for metastasis to the leptomeninges. BRAF inhibitors including vemurafenib, dabrafenib, and encorafenib have demonstrated clinical efficacy in the management of melanoma that harbor activating BRAF mutations (ie V600E) [131, 132]. While there are accumulating reports on response, it is difficult to determine at this time how much of an impact BRAF inhibitors have on the overall metastatic melanoma population [133–135]. BRAF inhibitors remain an option for patients with metastatic melanoma, but may not be as effective in patients with LMD that developed in patients already taking BRAF inhibitor therapy.

## Immunotherapies

Immunotherapy for LMD comes in many forms, including targeted passive immunotherapy (e.g., traztuzumab, discussed above), immune-checkpoint inhibition, and cellular therapy. Cancer cells may escape the host immune response by expressing immune checkpoint molecules, such as cytotoxic T-lymphocyte associated antigen 4 (CTLA-4) and programmed death ligand 1 (PD-L1), which block T-cell activation and proliferation. Immune-checkpoint inhibitors (ICIs) have been developed to target these signals and enhance the anti-cancer T-cell response. ICIs have proven effective in BM from advanced melanoma, metastatic kidney cancer, and metastatic non–small cell lung cancer [136–138] and interest has grown in their potential for treating LMD. A phase 2 trial found that PD-1 blockade with pembrolizumab achieved a CNS response rate of 38% in patients with solid tumor LMD, with two patients achieving complete response [139]. Two additional phase 2 trials from Dana-Farber, investigated pembrolizumab and combination ipilimumab/nivolumab in patients with solid tumor LMD [140, 141]. with mOS of 3.6 and 2.9 months, respectively. Importantly, these were single armed studies with grade 3 and above adverse event rates that are typical for patients receiving PDL1 or combination IO treatments, making the actual clinical benefit difficult to discern [140, 141].

Multiple immunotherapy trials for LMD are currently ongoing. One potential approach to increase the efficacy of ICIs for LMD is to combine the multi-VEGFR tyrosine kinase inhibitor (TKI) levatinib with pembrolizumab (NCT04729348). Chimeric antigen receptor (CAR)-T cells, where the patient’s own T cells are genetically engineered to target cancer, are also being developed to target HER-2 + breast LMD. Following preclinical studies in a mouse of model of metastatic breast cancer [6], HER-2 targeted CAR-T cells are being studied for breast LMD (NCT03696030). A trial of a bi-specific antibody (HER2Bi) armed activated T-cells (HER2 BATs) to target breast LMD is also underway (NCT03661424). Finally, IL13Rα2-CAR T cells are under investigation for patients with LMD from glioblastoma, ependymoma, or medulloblastoma (NCT04661384). One significant limitation of CAR-T therapy for LMD and other CNS neoplasia is that off-target inflammation can result in potentially devastating consequences including cerebral edema, obstructive hydrocephalus and increased intracranial pressure, patients receiving these therapies are typically closely monitored in an ICU setting [142].

### Intrathecal Therapy

Intrathecal (IT) chemotherapy can play an important role in the management of select patients with LMD. IT therapy is thought to avoid the cytotoxic side effects of systemic administration, especially myelosuppression; however, beyond risk of infection by penetration of this closed system, each agent carries its own potential side effect profile that may manifest as neurologic symptoms including encephalopathy, seizures, or focal neurological deficits [87, 143]. Appropriate patient selection is paramount, as the drug of choice must disseminate in the subarachnoid space to reach free-floating cancer cells and drug penetration into bulky disease is limited. When CSF flow is obstructed or the tumor nodules are > 2 mm, there is a limited role of IT therapy due to poor drug delivery and penetration [89].

The most common IT chemotherapeutic agents for LMD include methotrexate (MTX), thiotepa, and cytarabine [87, 89, 144]. There is no consensus regarding optimal drug dosing, frequency of administration, or duration of therapy. MTX and thiotepa are usually administered twice weekly for 4 weeks for induction, followed by every 2 weeks or monthly for consolidation and maintenance therapy [87, 89]. Liposomal cytarabine (i.e., Depocyt) was generally preferred over free cytarabine given its increased efficacy and extended half-life within the CSF, allowing for drug administration every 2 weeks; unfortunately, this agent is no longer commercially available due to technical issues within the manufacturing process [87]. While IT chemotherapy generally remains sequestered in the subarachnoid space, both IT MTX and thiotepa have been associated with systemic side effects including myelosuppression [87]. IT chemotherapy can also have neurologic complications, often presenting with headache, nausea, vomiting, or focal neurological signs [87, 89]. The most common neurological manifestation of IT chemotherapy complication is ventriculitis, either chemical or infectious, with reported incidence of 10–23% in patients treated with IT liposomal cytarabine [145, 146]. IT-MTX can have more devastating neurological consequences with a progressive leukoencephalopathy or transverse myelitis with increasing risk with cumulative MTX use, particularly in combination with radiation therapy [87, 89].

Although both MTX and ARA-C have long been treatment options for patients with LMD, the survival benefit remains questionable. Existing prospective studies show mOS of 14 to 16 weeks with mono-IT therapy, without additional benefit with addition of a second agent [147–150]. In a prospective study evaluating high-dose methotrexate in LMD from solid tumors, there was improved CSF cytologic clearing with high-dose IV MTX compared to IT-MTX at 81% and 60%, respectively; additionally, mOS in the high-dose MTX cohort was 13.8 months compared to 2.3 months in the IT-MTX cohort [151]. Other studies have also demonstrated limited survival benefit of IT chemotherapy with reports of mOS of 3–4 months compared to 6 months in those receiving systemic chemotherapy alone [6, 150, 152]. One open-label phase III trial found that combination therapy with IT-liposomal cytarabine and systemic therapy improved mOS compared to systemic chemotherapy alone in breast cancer patients with LMD (7.3 months vs. 4 months) [153]. The combination of lack-luster survival improvement along with side effects and frequent administration means IT chemotherapy with these traditional agents warrants careful consideration. In our practice, it is generally reserved for those who would otherwise not be able to tolerate systemic therapy or have no reasonable option for a CNS-active systemic therapy. It should be noted that use of high-dose IV MTX also requires careful patient selection based on functional status, motivation, and goals of care given that this typically requires multiple inpatient visits and close outpatient monitoring for potential toxicity.

There is growing data that IT pemetrexed, a multi-target anti-folate agent, may be efficacious in the management of LMD secondary to solid tumors with relatively low toxicity [154•, 155–157]. A phase I/II trial evaluating combination therapy of II pemetrexed with radiotherapy as first line CSF treatment in solid tumors found an adverse event rate of 53%, most of which were low grade [156]. mOS was 5.5 months with PFS of 3.5 months [156]. There are similar reports of mild AEs, most often myelosuppression, as well as mOS of up to 9.0 months in the literature [154•, 157].

### Intrathecal Immunotherapy

As we reviewed above, antibodies were historically thought to have limited activity in the CNS because of their large size. As a result, these highly effective biologics were good candidates for IT delivery. Trastusumab, pertuzumab, rituximab, and bevacizumab all appear to be relatively safe and better tolerated when administered IT than traditional chemotherapy, though chemical meningitis and headache do still occur.

Trastuzumab, a monoclonal antibody, is a form of passive immunotherapy that facilitates the uptake and recycling of HER2 receptors. In the early 2000s, just a few years after trastuzumab’s FDA approval, case reports of IT trastuzumab began to appear in the literature. These hinted at a safe and potentially effective therapy option for patients with HER2 + breast cancer LMD. A retrospective series of 18 individuals with breast LMD treated with single-agent IT trastuzumab were found to have a significant difference in 6-month PFS rates compared to those treated with IT chemotherapy or WBRT [98]. In another series of 13 patients treated with IT trastuzumab, 4 patients achieved sustained responses > 6 months [158]. Similarly, a systematic review of IT trastuzumab showed promising clinical, cytologic, and radiographic response rates [159]. A European phase 1 study did not identify any dose limiting toxicities due to IT trastuzumab and recommended 150 mg/week as their phase 2 dose [160], which in turn reported a mOS of 7.9mo in 19 patients [161]. Kumethekar et al., established the safety of an 80 mg twice weekly regimen, which demonstrated an mOS of 10.5 months in the phase II portion of the study [162•]. Taken together, these data suggest that IT trastuzumab appears to have better tolerability that traditional IT chemotherapy, and warrants further evaluation and consideration of use in select cases. Further studies are currently recruiting for the evaluation of IT trastuzumab in combination with pertuzumab for LMD secondary to HER2 + breast cancer (NCT04588545).

Interestingly, isolated case reports suggest that IT ICI directed at PD-1 in LMD may be safe, although the overall efficacy of these treatments remain unclear given the difficulty of running larger clinical studies in LMD [163]. Several phase 1 studies examining IT nivolumab and/or ipilimumab in leptomeningeal disease secondary to metastatic solid tumors are currently planned or ongoing (NCT03025256, NCT05112549, NCT05598853). Interestingly, in terms of IT cell-based immunotherapies, a small phase 1 study recently demonstrated ICV administration of GD-2 CAR-T cells for H3K27M-altered diffuse midline glioma led to less systemic toxicity and was associated with increased levels of pro-inflammatory cytokines and reduced immunosuppressive cells in the CSF, as compared to IV administration [142].

### Other Intrathecally Delivered Therapies

As previously discussed, there are multiple ongoing or planned clinical trials evaluating chemotherapy, small molecule inhibitors and immunotherapy with or without radiation. While many of the therapies discussed above are focused on treatment of LMD secondary to a particular malignancy type or sub-type, there are also ongoing studies evaluating cancer agnostic therapies for the treatment of LMD. The ongoing RESPECT-LM trial is evaluating the safety and efficacy of ICV delivery of Rhenium-186 Nanoliposomes for selective delivery of beta-emitting radiation in LMD secondary to NSCLC and breast cancer (NCT0503449). Depletion of iron in the CSF via deferoxamine has previously shown benefit in pre-clinical models of LMD [23••]; a phase 1a/1b study based at Memorial Sloan Kettering is now recruiting to evaluate the safety and efficacy of IT deferoxamine in patients with LMD secondary to any solid tumor (NCT05184816). Further studies involving generalizable treatments with efficacy across LMD subtypes are particularly important, given overall poor survival time from first LMD diagnosis and a dearth of effective novel treatments for LMD in solid tumors, hematologic malignancies and primary CNS tumors without known targetable mutations or available targeted therapy.

## Assessment of Treatment Response

Evaluating treatment response in LMD is an ongoing challenge and may contribute to LMD patients being excluded from clinical trials. Existing randomized trials utilize a variety of primary endpoints including mOS, PFS, neurological response rate, and time to neurologic response rate [87, 144, 147, 148, 164]. The complexity in standardizing LMD outcomes can be demonstrated by the difficulty the RANO LM working group has had in establishing a standard outcome measure. In 2017, the RANO Leptomeningeal Metastases Working Group published a consensus on treatment response for LMD, recommending consideration of CSF cytology, contrast enhancing MRI of the neuroaxis, and detailed neurological evaluation at regular intervals [165]. Just 2 years later, in 2019, the same group published a paper subtitled “lack of feasibility and clinical utility and a revised proposal,” highlighting the difficulty in standardizing treatment response evaluation for LMD [166]. In October 2022, the RANO LMD committee and EORTC brain tumor group released a new imaging scorecard [167••]. To date, none of these response criteria have been validated in a prospective interventional study of LMD. In fact, the recent pembrolizumab study by Brastianos et al. specifically chose not to use a surrogate endpoint, but chose to focus on overall survival [140]. As the sensitivity and specificity of the CSF liquid biopsy technologies become more defined, we anticipate that these will be incorporated into a comprehensive and clinically useful response criteria that includes imaging, CSF studies, clinical exam, and survival. In the meantime, we advocate that patients with LMD continue to be included in clinical trials, either in exploratory or CNS cohorts.

## Conclusions

LMD is a common complication of metastatic cancer, with devastating consequences in terms of overall mortality and morbidity. Diagnosis of LMD can be challenging, often requiring repetitive testing and resulting in substantial delay to treatment, further limiting our ability to extend duration and quality of life. Recent advances in diagnostics for LMD, including CTCs and cfDNA, may offer increased diagnostic sensitivity and shorten time to definitive diagnosis. While the overall prognosis of LMD remains poor across tumor types, recent studies of targeted small molecules, ADCs and IT based therapies for specific solid-tumor LMD have demonstrated an increase in overall and progression free survival, moving the needle further towards identification of durable treatment regimens for LMD.
